# Therapeutic Effect of P-Cymene on Lipid Profile, Liver Enzyme, and Akt/Mtor Pathway in Streptozotocin-Induced Diabetes Mellitus in Wistar Rats

**DOI:** 10.1155/2022/1015669

**Published:** 2022-04-26

**Authors:** Maryam Arabloei Sani, Parichehreh Yaghmaei, Zahra Hajebrahimi, Nasim Hayati Roodbari

**Affiliations:** ^1^Department of Biology, Science and Research Branch, Islamic Azad University, Tehran, Iran; ^2^A & S Research Institute, Ministry of Science Research and Technology, Tehran, Iran

## Abstract

Diabetes is a serious public health problem in low- and middle-income countries. There is a strong link between hyperglycemia, oxidative stress, inflammation, and the development of diabetes mellitus. PI3K/Akt/mTOR is the main signaling pathway of insulin for controlling lipid and glucose metabolism. P-cymene is an aromatic monoterpene with a widespread range of therapeutic properties including antioxidant and anti-inflammatory activity. In the present study, the antidiabetic effects of p-cymene were investigated. Diabetes was induced using streptozotocin in male Wistar rats. The effects of p-cymene and metformin were studied on levels of glucose (Glu), lipid profile, liver enzymes, oxidative stress, and the expression of Akt, phospho-Akt, and mTOR (mammalian target of rapamycin) proteins, using biochemical, histological, and immunohistochemical analysis. Data have shown that p-cymene can improve serum levels of Glu, total cholesterol (TC), triglycerides (TG), high-density lipoprotein cholesterol (HDL-c), low-density lipoprotein (LDL), very-low-density lipoprotein (VLDL), alkaline phosphatase (ALP), alanine aminotransferase (ALT), aspartate aminotransferase (AST), malondialdehyde (MDA), and the expression of mTOR, Akt, and phospho-Akt protein in diabetic animals. These results suggest that p-cymene has hypoglycemia, hypolipidemia, and antioxidant properties. It can regulate Akt/mTOR pathway and reduce hepatic and pancreas injury. It can be suggested for diabetes management alone or simultaneously with metformin.

## 1. Background

Type 2 diabetes mellitus (T2DM), also used to be called adult-onset diabetes, is the most common chronic metabolic disorder that is characterized mainly by high levels of glucose (Glu) concentration in the blood, resulting from insulin resistance and/or relative insufficient insulin secretion in peripheral tissues [1, 2]. Based on the reports, T2DM is the most frequent type of diabetes mellitus that accounts for 87% to 91% of all diabetes patients [3]. The World Health Organization estimated that 439 million people would have T2DM by the year 2030 [4]. As a multifactorial disease, T2DM development is caused by a combination of genetics and environmental factors, such as obesity, lack of physical activity, unhealthy diet, stress, cigarette smoking, and generous consumption of alcohol [5].

Extremely high blood glucose concentrations in T2DM people can lead to serious, potentially life-threatening vascular complications including atherosclerosis, retinopathy, neuropathy, nephropathy, and amputations [6]. A growing body of studies revealed an increase in biomarkers of oxidative stress in patients with T2DM, and especially in subjects with diabetes complications including micro- and macrovascular abnormalities [7–11]. As already mentioned, T2DM is a chronic metabolic disorder in which mitochondria have a key role as the most common source of ROS (reactive oxygen species) production. There is an important association between high levels of glucose in blood and induction of oxidative stress and inflammation on the one hand and the development of insulin resistance, T2DM, and its complications on the other. Hyperglycemia stimulates the production of ROS, the increased oxidative stress induces inflammation, and inflammation, in turn, increases the generation of ROS. Although many features of type 2 diabetes mellitus are not yet clear, it is revealed that enhanced production of ROS and inflammatory mediators have a central role in the development and progression of T2DM [12, 13]. Therefore, one strategy for T2DM therapy is controlling ROS production and using medication that improves insulin resistance.

There are a number of different types of diabetes drugs with some having similar ways of acting such as improving insulin resistance, controlling glucose levels, and reducing oxidative stress and inflammation [14–16]. However, most of these antidiabetic drugs have limited efficacy and many undesirable side effects such as drug resistance, weight gain, dropsy, and high rates of secondary failure [7, 10]. Therefore, there is a need for further development of low toxicity, effective and economic antidiabetic agents, and controlling T2DM complications, especially in long-term medication. In recent decades, using plant-based drugs has become popular in the world to treat many diseases instead of the use of chemical agents due to their minimal toxicity, easy access, cost-effectiveness, and easy use [17, 18]. In this regard, hypoglycaemic, antihyperlipidemic, antioxidant, and anti-inflammation effects of many monoterpenes have been reported in several experimental studies [19–21]. Monoterepens belong to the terpenoids group of secondary plant metabolites that are synthesized through the isoprenoid acid pathway. P-cymene is an aromatic organic monoterpene isolated from more than 100 various medicinal plants. A widespread range of therapeutic properties of p-cymene has been demonstrated including antioxidant, anti-inflammatory, antinociceptive, anxiolytic, anticancer, and antimicrobial effects [22–24].

With respect to the above description, the present study aims to investigate the effects of p-cymene on the prevention and treatment of T2DM in the rat model of diabetes. We found that p-cymene can improve serum levels of Glu, total cholesterol (TC), triglycerides (TG), high-density lipoprotein cholesterol (HDL-c), low-density lipoprotein (LDL), Alkaline phosphatase (ALP), alanine aminotransferase (ALT or SGPT), aspartate aminotransferase (AST or SGOT), malondialdehyde (MDA), and very-low-density lipoproteins (VLDL) in diabetic rats. Also, we analyzed the expression of mTOR, Akt, and phosphorylated Akt (phospho-Akt) protein. The mechanistic target of rapamycin (mTOR), also known as the mammalian target of rapamycin, is a serine/threonine-protein kinase in the phosphatidylinositol 3-kinase (PI3K-) related kinase (PIKK) family. It is regulated by the nutrient state, glucose, amino acids, and growth factors, so it plays an important role in the regulation of cell growth, cell proliferation, cell survival, protein synthesis, and metabolism of lipids and glucose [25]. Dysregulation in mTOR signaling is involved in several diseases such as obesity, type 2 diabetes mellitus, cancer, and neurodegenerative disorders [25]. Experimental studies suggested that obesity and overnutrition activate mTOR in various tissues in islets of humans [26, 27]. Akt or protein kinase B (PKB) is also a serine/threonine-protein kinase that regulates cellular processes including glucose metabolism, cell survival, and cell proliferation. Some reports have demonstrated that the Akt signaling pathway is associated with pathophysiological processes of diabetes mellitus and its complications [28].

## 2. Methods

### 2.1. Experimental Animals

Fifty-four male Wistar rats weighing between 200 and 250 g were purchased from Razi laboratory animal, Islamic Azad University, Science and Research Branch, Tehran, Iran. Animals were kept 4 per cage in the animal house of the Science and Research Branch of Islamic Azad University, under standard laboratory conditions of controlled room temperature (22°C) and humidity (50 ± 10%) with 12 hours of light and dark cycles before the experiment. Throughout the study period, the rat was allowed free access to water and standard chow. All efforts were made to avoid animal pain and suffering in accordance with the guidelines for the Care and Use of Laboratory Animals (Committee for the update of the guide for the Care and Use of Laboratory Animals, 1996). Applications were approved by the Animal Care and Use Committee of Islamic Azad University, Science and Research Branch. The animals were familiarized with the laboratory conditions for one week prior to starting the procedures. Diabetes was induced by a single intraperitoneal (i.p.) injection of 55 mg/kg streptozotocin (STZ) (Sigma-Aldrich, USA) freshly dissolved in 0.1 M sodium citrate buffer, pH 4.5 [29]. After 48 hours, blood samples were collected from the rat tail vein, and the amount of whole blood sugar was measured using a glucometer (Cera pet, South Korea). The animals with glucose levels greater than 300 mg/dl were considered diabetic and selected for further study. P-cymene was used at a dose of 25, 50, and 100, according to previous studies [22, 30].

In fact, animals were randomly divided into nine groups (*n* = 6) as follows: (1) the control group (C) that were fed with a standard diet and water ad libitumand received no treatment; (2) the sham operation group (*D*) or diabetic rats with single i.p. injection of 55 mg/kg STZ; (3) Metformin group (Met): diabetic rats with oral administration of metformin as a positive control of diabetic rats (55 mg/kg, Osve Pharmaceutical Co, Iran) for 4 weeks; (4) control-25 (C25): control rats treated with p-cymene (25 mg/kg, Sigma Chemical Co, St. Louis, MO, USA) for 4 weeks; (5) control-50 (C50): control rats treated with p-cymene (50 mg/kg) for 4 weeks; (6) control-100 (C100): control rats treated with p-cymene (100 mg/kg) for 4 weeks; (7) diabet-25 (D25): diabetic rats treated with p-cymene (25 mg/kg) for 4 weeks; (8) diabet-50 (D50): diabetic rats treated with p-cymene (50 mg/kg) for 4 weeks; (9) diabet-100 (D100): diabetic rats treated with p-cymene (100 mg/kg) for 4 weeks. The supplemented p-cymene was given by oral gavage. The body weights of animals were recorded at the beginning of the experiment and at the end of the experimental period.

### 2.2. Blood Sampling and Biochemical Analysis

Serum samples and liver tissues were collected at the end of the 28th day. For serum collecting, animals were anesthetized with ketamine (0.8 mg/kg, i.p.) and xylazine (0.5 mg/kg, i.p.). Blood samples were then collected from the left ventricle of the heart and kept at room temperature for 2 hours. Serum was obtained through centrifugation at 2500*g* for 5 min and then maintained at −20°C until subsequent analysis. The concentration of metabolic parameters such as TC, TG, HDL, LDL, ALP, ALT, AST, and VLDL was estimated by commercially available animal spectrophotometric assay kits (Pars Azmun Company, Tehran, Iran) according to the manufacturer's recommendations.

The method of Placer et al. was used to determine the amount of malondialdehyde (MDA) as an index of oxidative damage [31]. The assay is based on the reaction of MDA with thiobarbituric acid (TBA) and the production of a red compound with a maximum absorption peak at 532 nm. For this purpose, liver tissues were homogenized 1 : 10 with cold phosphate-buffered saline and centrifuged (14000*g*, 15 min, 4°C). The resulting supernatants were used for the assay of the MDA. Briefly, 250 *μ*l of homogenate was added to 500 *μ*l of a solution containing 15% trichloroacetic acid, 0.375% thiobarbituric acid, and 0.25N hydrochloric acid and placed in a boiling water bath for 10 min. The samples were cooled and centrifuged (3000 rpm, 10 min). Then, 200 *μ*l of the resulting supernatant was removed and quantified at 532 nm. Data were expressed as *μ*mol/l.

### 2.3. Histological Procedures, Dithizone Staining, and Immunohistochemistry

All animals were anesthetized at the end of the 4th week, and pancreatic tissues were excised and evaluated for standard histological procedures. Samples were trimmed free of fat and preserved in 10% paraformaldehyde and then embedded in paraffin after standard processing of dehydration in increasing concentration of ethanol and clearing in xylene. Then, paraffin samples were sectioned into the rotary microtome to obtain 5-6 *μ*m thick sections and then mounted on glass slides for dithizone (DTZ) staining. Dithizone is specific staining for a staining *B*-cell in pancreatic islets. It is a zinc-binding agent that selectively stains the islet's beta-cells and therefore turns the islets red. Beta-cells contain large amounts of zinc ions. Dithizone (Sigma-Aldrich) solution was prepared by dissolving 100 mg of dithizone in 10 mL dimethylsulfoxide (DMSO, Sigma-Aldrich). After 10 minutes, 40 ml PBS was added and filtered using a 0.2 *μ*m filter and used for staining tissue at 37°C for 15 minutes and the stained cells were observed under a light microscope [32].

Immunohistochemistry was done according to the standard protocol for immunohistochemistry, which was described everywhere [33–35]. Briefly, the sections of pancreatic tissue were cut at five micron-thick and placed on APES ((3-aminopropyl) triethoxysilane) coated microscopic slides for 1 hour at 37°C. Later, the tissues were deparaffinized with 2 changes of xylene, 5 minutes each, and rehydrated in 2 changes of 100% ethanol for 3 minutes each, 95% and 80% ethanol for 1 minute each. Then, they were rinsed in distilled water for 5 minutes. For antigen retrieval, slides were boiled in antigen retrieval solution (TBS 1X, Sigma-T5912, 20 min) and washed with PBS (3 times, 5 min, Sigma-P4417). Slides were rinsed in 0.3% triton for 30 minutes to permeabilize the cell membrane. Following PBS wash, the slides were blocked with 10% normal goat serum for 30 minutes. For detection of Akt, phospho-Akt, and mTOR protein, slides were incubated with primary mouse monoclonal anti-Akt1 antibody (1 : 100; sc-5298, Santa Cruz Biotechnology, Inc., United States), primary rabbit anti-phospho-Akt antibody (1 : 100; anti-phospho-Akt (ser473), ^#^4060, Cell Signaling Technology, Danvers, MA, USA), and mouse monoclonal anti-mTOR antibody (1 : 100; sc-517464, Santa Cruz Biotechnology, Inc., United States) at 4°C overnight, respectively. The next day, the sections were washed with PBS for 4 × 5 minutes. Later, slides were incubated with secondary antibody for 90 minutes at 37°C in the dark (for Akt and mTOR: FITC Goat Anti-Mouse (IgG) antibody, 1 : 150, ab6785, Abcam; and for phospho-Akt: FITC Goat Anti-Rabbit IgG (*H* + *L*) antibody, 1 : 150, orb688925, Biorbyt Ltd., USA). After four times washes, DAPI (Sigma-D9542) was added to slides and washed with PBS after 20 min. Images were observed using a Labomed microscope (USA). Quantification of the positive area was performed using ImageJ software (National Institute of Health, USA; https://imagej.nih.gov/ij).

### 2.4. Statistical Analysis

The data are presented as the means ± SEM One-Way ANOVA with Tukey's post hoc test, which was used for comparison among groups. All data were analyzed using IBM SPSS Statistics for Windows, version 20 (IBM Corp., Armonk, NY, USA). The charts were drawn using Microsoft Excel 2010. *p* < 0.05 was set as significant.

## 3. Results

### 3.1. Biochemical Results

Table 1 summarizes the serum levels of Glu, TG, TC, LDL, HDL, and VLDL in all animal groups. As indicated in Table 1, injection of STZ significantly increased the serum level of Glu, TG, TC, and VLDL in the sham group (*D*) in comparison to control animals. Administration of metformin or p-cymene improved Glu's, TG's, TC's, and VLDL's value in serum (in D25 and D100 groups for Glu, D25, D50, and D100 groups for TG; D25 and D50 groups for TC; and in D25, D50, and D100 groups for VLDL) when compared to the sham ones. Changes in Glu, TG, TC, and VLDL levels were most similar to the control group when treating diabetic animals with p-cymene at the dose of 25, 100, 25, and 25 or 50 mg/kg, respectively. It seems that the serum levels of LDL increased in the sham operation group but there was no significant difference between the control group and the sham group (*p* ≥ 0.05). Administration of metformin or p-cymene significantly decreased the blood level of LDL in C25, C100, and D100 groups (*p* ≤ 0.05) in comparison to the sham group. The serum levels of HDL decreased in the sham operation group but there was no significant difference between the control group and the sham group (*p* ≥ 0.05). Administration of metformin or p-cymene significantly increased the blood level of HDL in all groups (*p* ≤ 0.001) in comparison to the sham group, and in C25, C50, and C100 groups (*p* ≤ 0.001) when compared to control ones. P-cymene treatment did not have any effects on serum levels of Glu, TG, TC, and LDL in C25, C50, and C100 groups in comparison to controls (exception for Glu in the C50 group). In contrast, p-cymene treatment increased the levels of VLDL in C25, C50, and C100 animals when compared to control rats, but these increases were significantly lower compared to the VLDL level in the sham group.

The serum levels of AST, ALT, and ALP are presented in Table 2. We observed that injection of STZ significantly increased (*p* ≤ 0.001) the serum level of AST, ALT, and ALP in the sham group in comparison to control animals. Administration of metformin or p-cymene improved the serum level of these factors in diabetic rats (*p* ≤ 0.001) when compared to the sham group (except for AST in the D100 group; *p* ≥ 0.05). Changes in AST, ALT, and ALP levels were most similar to control animals when treating diabetic animals with p-cymene at the dose of 25, 25, and 50 mg/kg, respectively. P-cymene treatment did not have any effects (*p* ≥ 0.05) on serum levels of AST and ALT in C25, C50, and C100 groups in comparison to controls (except for AST in C25 group; *p* ≤ 0.01). In contrast, p-cymene treatment increased the levels of ALP in C25, C50, and C100 animals when compared to control rats (*p* ≤ 0.001), but these increases were significantly lower compared to the ALP levels in the sham group (*p* ≤ 0.001).

The levels of MDA in liver tissues are presented in Table 2. Injection of STZ markedly increased (*p* ≤ 0.001) the amount of MDA in the sham group in comparison to the control ones. Administration of metformin or p-cymene improved the amount of MDA in diabetic rats (*p* ≤ 0.001) when compared to the sham group. No difference was observed between the three groups of D25, D50, and D100.

### 3.2. Dithizone Staining of Pancreatic Tissues

**Figure 1:**
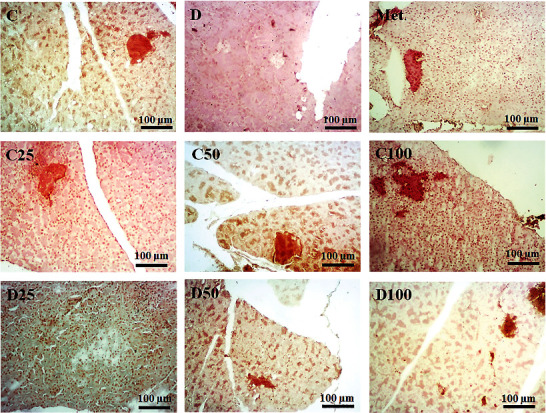
Dithizone staining of pancreatic tissues in all animal groups. The red or DTZ positive regions indicate the *B*-cells in islets of Langerhans. Administration of metformin or p-cymene obviously increased the *B*-cell mass in Met, D50, and D100 groups in a dose-dependent manner. C: control rats, D: the sham or diabetic rats, MET: diabetic rats + metformin (55 mg/kg), C25: control rats + p-cymene (25 mg/kg), C50: control rats + p-cymene (50 mg/kg), C100: control rats + p-cymene (100 mg/kg), D25: diabetic rats + p-cymene (25 mg/kg), D50: diabetic rats + p-cymene (50 mg/kg), and D100: diabetic rats + p-cymene (100 mg/kg). Magnification 400x Scale bar 100 *μ*m.

Zinc-binding substance diphenylthiocarbazone (dithizone or DTZ) was used to stain pancreatic tissues. Previous studies have demonstrated that DTZ staining does not adversely affect islet function either *in vitro* or *in vivo*.  Figure 1 shows the pancreatic tissues stained with the specific DTZ agent. The red or DTZ positive regions indicate the *B*-cells in islets of Langerhans with normal morphology and without significantly altering islets in the control, C25, C50, and C100 groups. DTZ staining showed loss of beta-cell mass of the Langerhans islets in the diabetic group (the sham or *D*). Administration of metformin or p-cymene improved the beta-cell mass of the Langerhans islets in diabetic rats (Met, D50, and D100) in a dose-dependent manner when compared to the sham group. Therefore, it seems that administration of p-cymene (100 mg/kg) may improve the changes of Langerhans islets in diabetic rats.

### 3.3. Immunohistochemical Analysis of Akt, phospho-Akt, and mTOR

**Figure 2:**
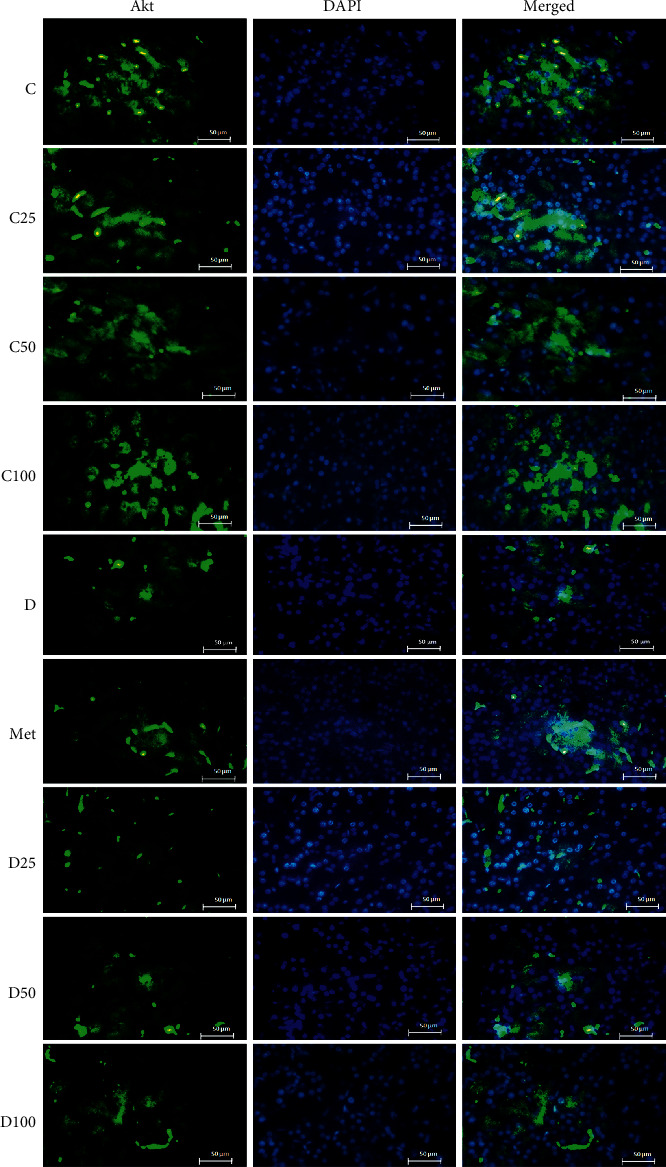
Fluorescence immunocytochemistry analysis of Akt. C: control rats, D: the sham or diabetic rats, MET: diabetic rats + metformin (55 mg/kg), C25: control rats + p-cymene (25 mg/kg), C50: control rats + p-cymene (50 mg/kg), C100: control rats + p-cymene (100 mg/kg), D25: diabetic rats + p-cymene (25 mg/kg), D50: diabetic rats + p-cymene (50 mg/kg), and D100: diabetic rats + p-cymene (100 mg/kg). Magnification 400x. Scale bar 50 *μ*m.

**Figure 3:**
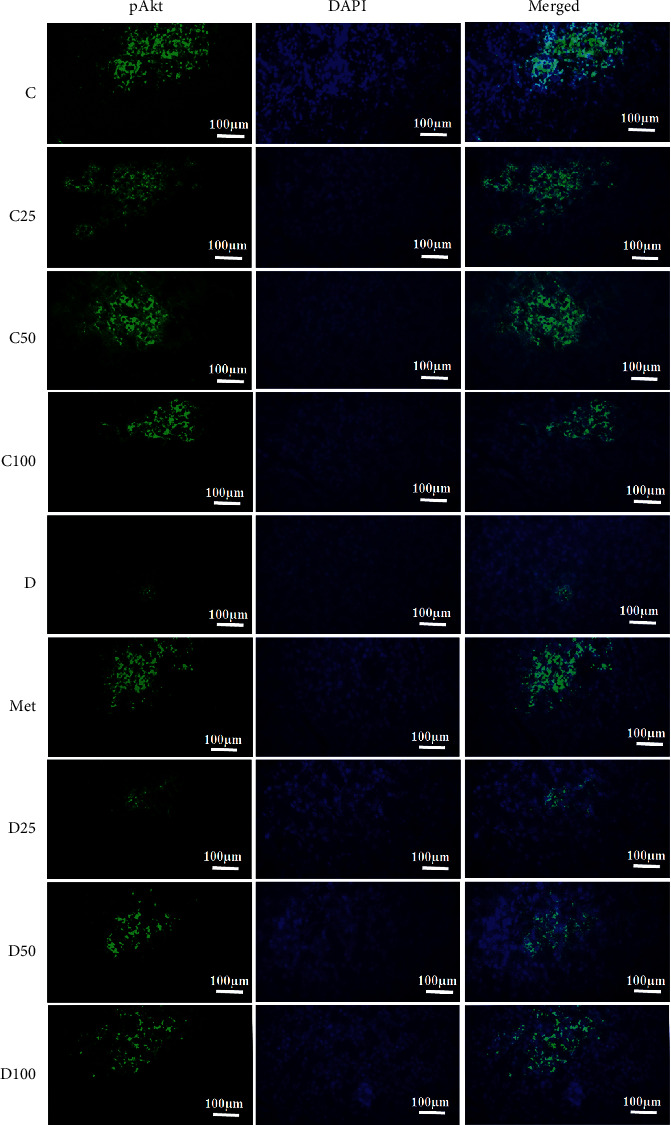
Fluorescence immunocytochemistry analysis of phospho-Akt. C: control rats, D: the sham or diabetic rats, MET: diabetic rats + metformin (55 mg/kg), C25: control rats + p-cymene (25 mg/kg), C50: control rats + p-cymene (50 mg/kg), C100: control rats + p-cymene (100 mg/kg), D25: diabetic rats + p-cymene (25 mg/kg), D50: diabetic rats + p-cymene (50 mg/kg), D100: diabetic rats + p-cymene (100 mg/kg), and pAkt: phospho-Akt. Magnification 400x. Scale bar 100 *μ*m.

**Figure 4:**
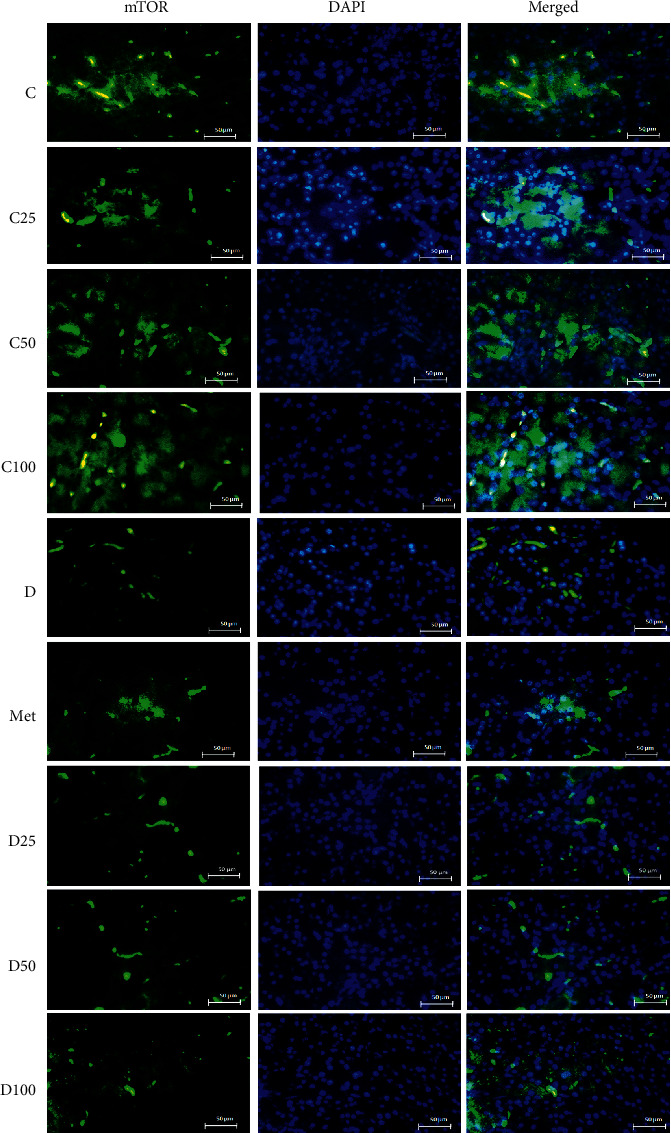
Fluorescence immunocytochemistry analysis of mTOR. C: control rats, D: the sham or diabetic rats, MET: diabetic rats + metformin (55 mg/kg), C25: control rats + p-cymene (25 mg/kg), C50: control rats + p-cymene (50 mg/kg), C100: control rats + p-cymene (100 mg/kg), D25: diabetic rats + p-cymene (25 mg/kg), D50: diabetic rats + p-cymene (50 mg/kg), D100: diabetic rats + p-cymene (100 mg/kg). Magnification 400x. Scale bar 50 *μ*m.

**Figure 5:**
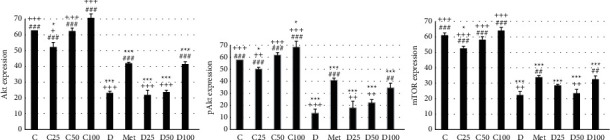
The expression of Akt, pAkt (phospho-Akt), and mTOR protein. ^*∗*^Statistically different from the control rats (*p* < 0.05), ^*∗∗∗*^statistically different from the control rats (*p* < 0.001), ^##^statistically different from the sham rats (*p* < 0.01), ^###^statistically different from the sham rats (*p* < 0.001), ^+^statistically different from the Met rats (*p* < 0.05), ^++^statistically different from the Met rats (*p* < 0.01), ^+++^statistically different from the Met rats (*p* < 0.001). C: control rats, D: the sham or diabetic rats, MET: diabetic rats + metformin (55 mg/kg), C25: control rats + p-cymene (25 mg/kg), C50: control rats + p-cymene (50 mg/kg), C100: control rats + p-cymene (100 mg/kg), D25: diabetic rats + p-cymene (25 mg/kg), D50: diabetic rats + p-cymene (50 mg/kg), and D100: diabetic rats + p-cymene (100 mg/kg).

Figures 2–5 show the level of Akt, phospho-Akt, and mTOR protein in the pancreas of rats in all animal groups, respectively. As indicated in Figure 5, the level of Akt, phospho-Akt, and mTOR protein in the control groups (control, C25, C50, and C100) was markedly higher than that in the diabetic groups (the sham, Met, D25, D50, and D100). Injection of STZ significantly reduced the expression of Akt, phospho-Akt, and mTOR proteins in the sham group in comparison to control animals. Data showed that the decrease in phospho-Akt in the diabetic group (the sham or *D*) was much greater than the decrease in Akt. Administration of metformin or p-cymene (100 mg/kg) increased Akt's, phospho-Akt's, and mTOR's expression in Met and D100 groups, respectively. The increase in phospho-Akt was much greater than the increase in Akt. There was no significant difference in Akt's, phospho-Akt's, and mTOR's expression between the sham, D25, and D50 groups.

## 4. Discussion

In the present study, we found that p-cymene ameliorates some pathophysiological features in a Wistar rat model of diabetes. Data have shown that p-cymene can improve serum levels of Glu, TC, TG, HDL-c, LDL, ALP, ALT, AST, MDA, and VLDL in diabetic rats. Also, it improved the expression of mTOR, Akt, and phospho-Akt protein in diabetic animals. Intraperitoneal injection of streptozotocin was used for the experimental developing models of diabetes mellitus. Many studies have utilized streptozotocin for the induction of diabetes models in animals. This model is based on preferential necrosis of beta-cells by streptozotocin resulting in hypoinsulinemia and hyperglycemia. Streptozotocin is an antimicrobial compound extracted from the bacterium *Streptomyces achromogenes* that selectively damages *B*-cells in pancreatic islets [36]. It is structurally similar to a glucose molecule; therefore, it can bind to the glucose transport (GLUT2) receptor and enter the cell. Pancreatic beta-cells are rich in GLU2 receptors, so they are a specific choice for streptozotocin [36]. In the present study, DTZ staining showed a loss of beta-cell mass of the Langerhans islets in the streptozotocin-induced diabetic group. Based on the obtained data, extensive pathological changes, such as hyperglycemia, hyperlipidemia, increased activities of liver enzymes, and increased oxidative stress were observed in the diabetic group. The serum levels of TC, TG, and VLDL were increased in the diabetic rats in comparison to control ones, indicating hyperlipidemia. Hyperlipidemia is very common in subjects with diabetes mellitus which is one of the reasons for the high risk of coronary heart disease in these individuals [37]. Hyperlipidemia is a result of a decrease in the activity of the lipoprotein lipases in patients with diabetes and hypoinsulinemia conditions [38]. Increased AST, ALT, and ALP were also observed in the serum of the diabetic group in comparison to control rats. AST, ALT, and ALP act as markers of liver function and their increase indicates liver injury [39]. Previous reports have shown that the serum levels of ASP, ALT, and ALP increase in patients with diabetes, insulin resistance, and metabolic syndrome [40–42]. Because of the central role of the liver in the homeostasis of glucose, hypoinsulinemia affects the liver and leads to hepatic injury [43]. The mechanism of induction of liver damage by streptozotocin is not well understood. Zafar et al. [44] showed elevated liver enzymes such as AST, ALT, and ALP following streptozotocin treatment. Also, they showed accumulation of lipid droplets, lymphocytic infiltration, and increased fibrous content in the liver of treated animals. They suggested that diabetic complications in the liver may be the result of changes in liver enzymes.

Liver tissues analysis showed an increase in oxidative stress in STZ-induced diabetic rats. Many studies revealed an increase in biomarkers of oxidative stress in patients with diabetes [7–11]. Diabetes is a chronic metabolic disorder in which mitochondria have a key role as the most common source of ROS production. There is an important association between high levels of glucose in the blood and the induction of oxidative stress [12, 13]. Therefore, one strategy for T2DM therapy is controlling ROS production. MDA is a known oxidative stress biomarker that results from lipid peroxidation of polyunsaturated fatty acids by ROS [45]. In the current study, injection of STZ markedly increased the amount of MDA in the sham rats which indicated oxidative stress in diabetic animals.

The expressions of mTOR, Akt, and phospho-Akt proteins were determined in the diabetic animals, too. The PI3K/Akt/mTOR pathway is crucial in the regulation of signal transduction and many cellular mechanisms including survival, proliferation, growth, metabolism, angiogenesis, and metastasis, in both normal and pathological conditions. Dysregulation of this pathway is associated with many human disorders including diabetes [46]. Activation of PI3K results in Akt phosphorylation, Akt activity, and Akt recruiting to the cell membrane. Phosphorylated Akt mediates the phosphorylation of mTOR to activate it, which subsequently regulates the growth and metabolism of glucose and lipid [47, 48]. Among the various factors that are identified to enhance the PI3K/Akt pathway, insulin is a crucial activator [49]. Therefore, changes in insulin can affect Akt's activity. Both decreased and increased Akt's function has been reported in diabetes mellitus [28]. In the present study, the expression of Akt, phospho-Akt, and mTOR proteins was decreased in the pancreatic tissues of diabetic animals that are in line with the results of Bathina and Das [50]. Bathina and Das examined the alteration of the PI3K/Akt/mTOR pathway in the brain of streptozotocin-induced type 2 diabetes rats. They have shown that oxidative stress and apoptosis of pancreatic beta-cells can be increased following treatment with streptozotocin. They also showed that streptozotocin can reduce the expression of phosphorylated Akt and phosphorylated mTOR in treated animals [50]. In the present work, the decrease in phospho-Akt in the diabetic group (the sham or *D*) was much greater than the decrease in Akt. Therefore, streptozotocin may block signal transduction via dysregulation of the PI3K/Akt/mTOR pathway, which is subsequently associated with pathophysiological processes of diabetes mellitus and its complications.

Here, we investigated the effects of p-cymene on the treatment of T2DM in a rat model of diabetes and we compared its effects with metformin. P-cymene [1-methyl-4-(1-methylethyl)-benzene] is an aromatic organic monoterpene isolated from more than 100 various medicinal plants which belong to the *Thymus* genus. A widespread range of pharmaceutical properties of p-cymene has been demonstrated including antioxidant, anti-inflammatory, antinociceptive, anxiolytic, anticancer, and antimicrobial effects [22–24]. Metformin is a first-line medicine to control high blood glucose in patients with type 2 diabetes mellitus. It reduces the amount of glucose absorbed from food and the amount of glucose produced by the liver. Also, it increases the response of the body to insulin [51]. In the present study, p-cymene was used at three doses including 25, 50, and 100 mg/kg. Administration of p-cymene in the current study ameliorated adverse features induced by streptozotocin. P-cymene treatment resulted in a significant decrease in serum glucose levels of diabetic rats in a similar way to metformin, which is in line with the results of Lotfi et al. [30]. They showed that p-cymene and metformin, alone or in combination, can decrease the blood amounts of glucose in mice with a high-fat diet [30]. Similar results were reported in studies by Ghazanfar et al. [52] and Bayramoglu et al. [53]. They suggested antidiabetic and blood-glucose-lowering properties of *Artemisia amygdalina* extracts and Oregano oil in STZ-induced diabetic rats [52, 53]. One of the active components in these extracts is p-cymene. Also, we observed that glucose level was increased by the p-cymene treatment in the control rats, although overall not significantly. The only significant effect on glucose levels in control rats was observed by the use of 50 mg/kg p-cymene. A similar result was observed in the D50 group. Although p-cymene decreased the amount of glucose in diabetic groups, this reduction was much greater in D25 and D100 groups than in the D50 group. It seems that low and high doses of p-cymene can improve the glucose level in streptozotocin-induced diabetic rats, and the median doses of it (50 mg/kg) have the opposite effect on the blood glucose level.

The lipid profile was also influenced by the p-cymene treatment. Data analyses have shown a positive effect of the p-cymene on lipid factors including TG, TC, and VLDL in diabetic animals, which are in line with the results of Lotfi et al. [30], Ghazanfar et al. [52], and Bayramoglu et al. [53]. Changes in TG, TC, and VLDL levels were most similar to the control group when treating diabetic animals with p-cymene at the dose of 100, 25, and 25 or 50 mg/kg, respectively. However, administration of p-cymene improved TG's, TC's, and VLDL's value in serum of all diabetic rats (exception for TC in the D100 group) when compared to the sham ones. Therefore, it seems that p-cymene at a dose of 25 and 50 mg/kg is much more effective than p-cymene at a dose of 100 mg/kg. Streptozotocin had no effect on blood levels of HDL; however, p-cymene increased the blood HDL in all treated animals (C25, C50, C100, D50, and D100 groups). With respect to the p-cymene effect on lipid profile, since a decrease was observed in TG, TC, and VLDL amount in treated groups in comparison to the sham group, and an increase was observed in HDL amount in treated animals in comparison to control one, the hypolipidemic potential of p-cymene could not be discarded, but further experiment with other suitable animal models of diabetic needs to be done.

The profile of the liver enzyme was also influenced by the p-cymene administration in a similar way to the metformin. Administration of metformin or p-cymene markedly decreased the rate of AST, ALT, and ALP in the blood of streptozotocin-induced diabetic (D25, D50, and D100 groups) animals, except for AST in the D100 group. Therefore, it seems that p-cymene can prevent beta-cell destruction and can reduce liver injury in streptozotocin-induced diabetic rats. Similar results were also observed in studies by Lotfi et al. [30], Ghazanfar et al. [52], and Bayramoglu et al. [53]. Changes in AST, ALT, and ALP levels were most similar to control animals when treating diabetic animals with p-cymene at the dose of 25, 25, and 50 mg/kg, respectively. Therefore, it seems that p-cymene at a dose of 25 and 50 mg/kg is much more effective than p-cymene at a dose of 100 mg/kg. ALP level was increased by the p-cymene treatment in the control rats (C25, C50, and C100 group) although its increase was much lower compared to the sham rats. This observation may limit the administration of p-cymene in a healthy population. Additional studies are needed to fully distinguish this observation.

P-cymene treatment also decreased oxidative stress in diabetic animals. Administration of p-cymene significantly decreased MDA levels in the diabetic groups, indicating antioxidant properties of p-cymene. The antioxidant activity of p-cymene was suggested by Oliveira et al. [22]. No difference was observed between the three doses of 25, 50, and 100 mg/kg of p-cymene.

The effect of p-cymene on the expression of Akt, phospho-Akt, and mTOR protein was also examined. Our experiments have shown a positive but small effect of metformin or p-cymene on the expression of Akt, phospho-Akt, and mTOR protein in streptozotocin-induced diabetic animals. In fact, the expression of Akt, phospho-Akt, and mTOR protein decreased in diabetic animals, but after metformin or p-cymene treatment, the amount of these proteins increased moderately. The increase in phospho-Akt was much greater than the increase in Akt. In addition, this increase was much greater at the dose of 100 mg/kg of p-cymene, suggesting that p-cymene affects Akt/mTOR signaling pathway in a dose-dependent manner. No difference was observed between the two doses of 25 and 50 mg/kg of p-cymene.

## 5. Conclusions

Overall, this study showed that hyperglycemia, hyperlipidemia, injury of the liver, oxidative stress, and suppression of the Akt/mTOR signaling pathway occur in streptozotocin-induced diabetes rats. Administration of p-cymene significantly prevented the progression of diabetes. It probably has promising antidiabetic potential and can reduce liver injury and oxidative stress and can improve Akt/mTOR signaling pathway. According to the results, p-cymene may be suggested for the control of diabetes in diabetic individuals. However, the effective dose, period of treatment, and interaction with other supplements must be investigated. Further studies are required to investigate the mechanism responsible for the antidiabetic characteristic of p-cymene. The antidiabetic effects of p-cymene are comparable with metformin and may be used as adjunct treatments for diabetic patients.

## Figures and Tables

**Table 1:** Biochemical results of the diabetes rats and controls.

Group	Glu (mg/dl)	TG (mg/dl)	TC (mg/dl)	LDL (mg/dl)	HDL (mg/dl)	VLDL (mg/dl)
Control (*C*)	156.50 ± 11.80^###^	79.83 ± 3.19^+++#^	61.67 ± 2.22^+##^	18.67 ± 2.22	34.33 ± 1.89	7.55 ± 0.58^###^
Sham (*D*)	317.00 ± 18.01^*∗∗∗*^^+++^	101.33 ± 5.50^*∗*^^+++^	78.83 ± 3.18^*∗∗*^^+++^	22.67 ± 1.33^++^	28.50 ± 1.75^+++^	23.00 ± 1.53^*∗∗∗*^^+++^
Met	161.00 ± 18.88^###^	36.67 ± 2.65^*∗∗∗*^^###^	48.50 ± 3.92^*∗*^^###^	15.50 ± 1.15^##^	44.33 ± 2.50^###^	7.62 ± 0.70^###^
C25	173.83 ± 16.09^###^	81.33 ± 6.16^+++^	57.83 ± 2.61^###^	16.17 ± 0.54^#^	53.17 ± 4.80^*∗∗∗*^^###^	14.60 ± 0.61^*∗∗∗*^^###+++^
C50	232.00 ± 26.97	87.17 ± 2.68^+++^	71.50 ± 2.17^+++^	18.83 ± 1.25	60.83 ± 1.08^*∗∗∗*^^###+++^	16.33 ± 1.22^*∗∗∗*^^###+++^
C100	191.17 ± 15.37^###^	67.67 ± 5.23^+++###^	66.50 ± 1.95^+++^	16.83 ± 1.30^#^	52.5 ± 2.03^*∗∗∗*^^###^	12.38 ± 0.73^*∗∗*^^###++^
D25	207.67 ± 13.53^##^	37.00 ± 2.66^*∗∗∗*^^###^	58.00 ± 2.35^###^	18.50 ± 1.26	44.0 ± 1.37^##^	7.80 ± 0.60^###^
D50	233.00 ± 20.23	38.00 ± 2.79^*∗∗∗*^^###^	65.83 ± 3.46^#++^	18.67 ± 0.33	47.67 ± 2.08^*∗∗*^^###^	7.32 ± 0.52^###^
D100	205.50 ± 29.34^##^	64.83 ± 8.09^++###^	68.17 ± 2.83^+++^	17.00 ± 0.86^#^	45.83 ± 2.52^*∗*^^###^	13.03 ± 0.80^*∗∗*^^###++^

Values are presented as mean ± SEM(*n* = 6/each group).^*∗*^Statistically different from the control rats (*p* < 0.05), ^*∗∗*^statistically different from the control rats (c), ^*∗∗∗*^statistically different from the control rats (*p* < 0.001), ^#^statistically different from the sham rats (*p* < 0.05), ^##^statistically different from the sham rats (*p* < 0.01), ^###^statistically different from the sham rats (*p* < 0.001), ^+^Statistically different from the Met rats (*p* < 0.05), ^++^statistically different from the Met rats (*p* < 0.01), ^+++^statistically different from the Met rats (*p* < 0.001). The sham (*D*): diabetic rats, MET: diabetic rats + metformin(55 mg/kg), C25: control rats + p-cymene (25 mg/kg), C50: control rats + p-cymene (50 mg/kg), C100: control rats + p-cymene (100 mg/kg), D25: diabetic rats + p-cymene (25 mg/kg), D50: diabetic rats + p-cymene (50 mg/kg), D100: diabetic rats + p-cymene (100 mg/kg). TG: Triglyceride, TC: Total Cholesterol, LDL: low-density lipoprotein, HDL: high-density lipoprotein, VLDL: very low-density lipoproteins.

**Table 2:** Results of the AST, ALT, ALP, and MDA in diabetes rats and controls.

Group	AST (mg/dl)	ALT (mg/dl)	ALP (mg/dl)	MDA (*μ*mol/l)
Control (*C*)	81.50 ± 2.53^###^	66.17 ± 4.10^###^	473.80 ± 26.11^###^	5.17 ± 0.40^###^
Sham (*D*)	110.67 ± 1.48^*∗∗∗*^^+++^	97.17 ± 1.17^*∗∗∗*^^+++^	1007.50 ± 16.01^*∗∗∗*^^+++^	10.17 ± 0.48^*∗∗∗*^^+++^
Met	92.00 ± 2.83^###^	57.00 ± 2.84^###^	391.67 ± 14.40^###^	5.25 ± 0.48^###^
C25	95.25 ± 1.28^*∗∗*^^###^	65.33 ± 3.38^###^	777.75 ± 29.58^*∗∗∗*^^##+++^	5.83 ± 0.31^###^
C50	92.17 ± 2.77^###^	72.83 ± 4.73^###^	741.20 ± 27.94^*∗∗∗*^^###+++^	5.33 ± 0.21^###^
C100	91.33 ± 2.09^###^	56.50 ± 4.51^###^	730.50 ± 36.38^*∗∗∗*^^###+++^	5.00 ± 0.37^###^
D25	87.00 ± 2.32^###^	66.00 ± 6.52^###^	500.33 ± 52.85^###^	6.67 ± 0.33^###^
D50	94.50 ± 1.80^*∗∗*^^###^	59.00 ± 2.00^###^	457.50 ± 48.08^###^	7.00 ± 0.37^*∗*^^###+^
D100	102.33 ± 2.08^*∗∗∗*^^+^	58.83 ± 1.14^###^	523.20 ± 33.68^###^	6.63 ± 0.33^###^

Values are presented as mean ± SEM (*n* = 6/each group). ^*∗∗*^Statistically different from the control rats (*p* < 0.01), ^*∗∗∗*^statistically different from the control rats (*p* < 0.001); ^##^statistically different from the sham rats (*p* < 0.01), ^###^statistically different from the sham rats (*p* < 0.001); ^+^Statistically different from the Met rats (*p* < 0.05), ^+++^statistically different from the Met rats (*p* < 0.001). The sham (*D*): diabetic rats, MET: diabetic rats + metformin (55 mg/kg), C25: control rats + p-cymene (25 mg/kg), C50: control rats + p-cymene (50 mg/kg), C100: control rats + p-cymene (100 mg/kg), D25: diabetic rats + p-cymene (25 mg/kg), D50: diabetic rats + p-cymene (50 mg/kg), D100: diabetic rats + p-cymene (100 mg/kg). AST: aspartate aminotransferase, ALT: alanine aminotransferase, ALP: alkaline phosphatase, MDA: malondialdehyde.

## Data Availability

Data for the present study was generated during the study.
